# Dissemination of Spotted Fever Rickettsia Agents in Europe by Migrating Birds

**DOI:** 10.1371/journal.pone.0008572

**Published:** 2010-01-05

**Authors:** Karin Elfving, Björn Olsen, Sven Bergström, Jonas Waldenström, Åke Lundkvist, Anders Sjöstedt, Hans Mejlon, Kenneth Nilsson

**Affiliations:** 1 Clinical Bacteriology, Department of Medical Sciences, Uppsala University Hospital, Uppsala, Sweden; 2 Infectious Diseases, Department of Medical Sciences, Uppsala University Hospital, Uppsala, Sweden; 3 Section for Zoonotic Ecology and Epidemiology, Kalmar University, Kalmar, Sweden; 4 Department of Molecular Biology, Umeå University, Umeå, Sweden; 5 Swedish Institute for Infectious Disease Control, Solna, Sweden; 6 Clinical Bacteriology, Department of Clinical Microbiology, Umeå University Hospital, Umeå, Sweden; 7 Museum of Evolution, Uppsala University, Uppsala, Sweden; 8 Center of Clinical Research, Dalarna, Falun, Sweden; 9 Department of Clinical Microbiology, Falu Hospital, Falun, Sweden; University of Swansea, United Kingdom

## Abstract

Migratory birds are known to play a role as long-distance vectors for many microorganisms. To investigate whether this is true of rickettsial agents as well, we characterized tick infestation and gathered ticks from 13,260 migratory passerine birds in Sweden. A total of 1127 *Ixodes* spp. ticks were removed from these birds and the extracted DNA from 957 of them was available for analyses. The DNA was assayed for detection of *Rickettsia* spp. using real-time PCR, followed by DNA sequencing for species identification. *Rickettsia* spp. organisms were detected in 108 (11.3%) of the ticks. *Rickettsia helvetica*, a spotted fever rickettsia associated with human infections, was predominant among the PCR-positive samples. In 9 (0.8%) of the ticks, the partial sequences of 17kDa and *omp*B genes showed the greatest similarity to *Rickettsia monacensis*, an etiologic agent of Mediterranean spotted fever-like illness, previously described in southern Europe as well as to the *Rickettsia* sp.IrITA3 strain. For 15 (1.4%) of the ticks, the 17kDa, *omp*B, *glt*A and *omp*A genes showed the greatest similarity to *Rickettsia* sp. strain Davousti, *Rickettsia japonica* and *Rickettsia heilongjiangensis*, all closely phylogenetically related, the former previously found in *Amblyomma tholloni* ticks in Africa and previously not detected in *Ixodes* spp. ticks. The infestation prevalence of ticks infected with rickettsial organisms was four times higher among ground foraging birds than among other bird species, but the two groups were equally competent in transmitting *Rickettsia* species. The birds did not seem to serve as reservoir hosts for *Rickettsia* spp., but in one case it seems likely that the bird was rickettsiemic and that the ticks had acquired the bacteria from the blood of the bird. In conclusion, migratory passerine birds host epidemiologically important vector ticks and *Rickettsia* species and contribute to the geographic distribution of spotted fever rickettsial agents and their diseases.

## Introduction

The spotted fever group (SFG) rickettsiae are obligate intracellular bacteria transmitted by various arthropods species, which are either vectors and/or reservoirs for the organisms [1]. The SFG comprises about 20 different species, of which 14 are well-characterized pathogens [1]. The members of SFG, which have a worldwide distribution, are mainly associated with ticks, but also with fleas and mites [2], and the distribution of the rickettsioses will be identical to that of its competent arthropod vector when the tick serves as a reservoir.

Although rickettsial species are principally associated with arthropods, they are also capable of infecting vertebrates, including humans, in most cases as accidental hosts when bitten by ticks. The common clinical symptoms include fever, rash, muscle pain, headache, lymphadenopathy and a characteristic inoculation eschar at the bite site [3].

*Rickettsia helvetica* is the only spotted fever rickettsia (SFR) reported from Sweden, and documented patients have presented a mild, self-limited disease associated with fever, headaches and myalgias, but an acute febrile clinical picture has also been described [4], [5]. In two cases, however, there is also a documented association with perimyocarditis and sudden death [6].

Migratory birds, can act as long-distance vectors for several microbial agents of human disease, including viruses, chlamydiae, enterobacteria, and Lyme borreliosis (LB) spirochetes [7]–[11], and are also incidentally reported for rickettsiae [12].

Of the wide range of tick species that have been reported to parasitize wild birds, *Ixodes* and argasid ticks are the most frequent. However, as a vector and reservoir for SFG rickettsiae, ticks belonging to the family Ixodidae are the most important [1]. Ticks may acquire rickettsiae during blood meal acquisition from a rickettsiaemic host, like small mammals, or through transovarial passage from adult female ticks to the subsequent generation of ticks [13]. Ixodid ticks feeds only once at each life stage and rickettsiae can be transmitted to another host at the next blood meal.

Questions regarding the reservoir competence of birds and other vertebrate populations are unresolved or not fully understood with respect to SFR. In some natural vertebrate hosts, infections may result in rickettsemia that allow non-infected ticks to be infected, as compared to other animals that produce rickettsemias at too low levels or too transiently to infect ticks [14]. The contribution of birds in hosting and infecting ticks is important to examine, as are the epidemiological and clinical aspects of rickettsiae associated with birds.

In the current investigation, we characterized the tick infestation of and *Rickettsia* spp. transmission caused by migratory passerine birds captured in southern Sweden. The study is part of an investigation examining several vector-borne agents in the same tick material, in which presence of *Borrelia* spp. and TBE-virus previously have been reported [15], [16]. Our aim was to further define their role as disseminators of *Rickettsia* spp. infected arthropod vectors as well as their role as potential reservoir of the agents.

## Results

### Birds and Tick Infestation

13,260 migratory birds were checked for tick infestation from March 17 to November 13, 2001. Overall, 1127 ticks were originally collected from a total of 437 infested birds (2.1–2.6 ticks per infested bird) with predominance of sub-adult tick stages. All ticks were separately stored in snap-lid tubes and frozen at −70°C. They were later with a dissecting microscope identified to species and stage of development. Because the tick samples had been used for different analyses, extracted DNA from 957 of these ticks representing 529 larvae, 409 nymphs and 19 undetermined or adults collected from 407 birds was available for analyses of *Rickettsia* spp. (Table S1). Most of these 957 ticks were identified as *I. ricinus*, and 25 were partly damaged and only characterized as *Ixodes* spp. Four nymphs, from one bird, were identified as *I. lividus*. Of the 957 analysed ticks, 216 (22.8%) were collected during spring and 741 of 957 (77.4%) during autumn migration (Table 1). Of the birds, 111 (27.3%) and 297 (72.7%) of 407 were captured during spring and autumn migration, respectively.

**Table 1:** *Rickettsia* spp. in *Ixodes ricinus* ticks from migratory birds.

	Larvae	Nymphs	Tick(UD) or adults	Total
		AM/SM		AM/SM		AM/SM		AM/SM
Total no. of ticks tested	529	468/61	409	263/146	19	10/9	957	741/216
No (%) found positive:	36 (6.8)	19/17	68 (16.6)	20/48	4	1/3	108	40/68
*R. helvetica* 1	27	16/11	28	11/17	2	0/2	57	27/30
Other SFR^1^	2	0/2	20	5/15	2	1/1	24	6/18
Undetermined Rickettsia	7	3/4	20	4/16	0	0/0	27	7/20

1 *Rickettsia* species was determined for 81 of the 108 *glt*A-positive samples by sequencing partial regions of the 17kDa, *omp*B, *omp*A and citrate synthase genes (*glt*A).

AM = Autumn migration. SM = Spring migration. SFR = Spotted fever rickettsia.

UD = Undetermined (only detected in real-time PCR though no additional extracted sample was available for nested PCR).

The number of ticks per infested bird was between 1–41, and infestation with sub-adult stages was about 3-fold more common among ground-foraging birds, which also carried 82% of all analysed ticks.

### *Rickettsia* spp. Infection of Ticks

We determined the prevalence of *Rickettsia* spp. infection in collected larvae and nymphs using real-time PCR. All individual positive samples were rerun using nested PCR to obtain readable sequence data and identify individual species. One hundred and eight of the 957 (11.3%) ticks, collected from 77 of the 407 (18.9%) birds, were positive for *Rickettsia* spp., as detected using real-time PCR. Sixty-eight of the 108 (63%) ticks collected from birds during spring migration and 40 of the 108 (37%) collected during autumn migration were positive samples (Table S1).

Two hundred and fifteen birds were infested with one or more larvae. Of these, 127 were infested with one and 86 with two or more larvae. The prevalence of *Rickettsia* infection in larvae was 9 of 127 (7.1%) birds among the single infested birds compared to 22 of 86 (25.6%) infected larvae among the multiple infested birds. The higher prevalence of infection in larvae among multiple then in the single infested birds could be an indication of either reservoir competence among birds or transmission between ticks via a co-feeding mechanism [OR = 4.50; CI 2.0–10.4].

Another way to differentiate between these alternative explanations was to compare the count of infected larvae at 7.1% prevalence of infection (as for larvae from single-infested birds) with the observed prevalence of infection after the first positive larva had been identified. Twenty-two birds were multiple infested with 102 larvae and 28 infected larvae were found. The expected count of positive larvae was (102−22)×7.1% = 5.7 larvae, compared to the 6 observed, which does not support the reservoir competence explanation. However, one Blue Tit (*Parus caeruleus*) captured in November, carried 7 ticks of which 6 (Table S1 [Samples L88–89 = *I. ricinus*, L91 = undetermined, L 92–94 = *I. lividus*] were infected with *Rickettsia* spp. that turned out to belong to the *R. heilongjiangensis* group. Likewise was the Blue Tit and Redwing Trush (*Turdus iliacus*), from the spring migration, both infested with two infected nymphs at the same time. When comparing the total number of birds infested with infected ticks compared to the total number of collected birds, the figures were 59/6174 = 1% in ground-foraging birds and 18/7085 = 0.25% in other birds indicating that the former group of birds has a higher risk of exposure to ticks as a result of their feeding habits [Fisher's exact test, p<0.001].

To compare the frequency of infection among birds in the two groups, we measured the ratio of birds with infected larvae to the number of larvae-infested birds. Twenty-three (14.5%) of the 165 ground foragers and 8 (16%) of the 50 birds from the other group carried infected larvae, resulting in no difference in infection ratio between the groups [OR = 1.2; CI 0.5–2.8].

### *Rickettsia* Species Composition

Eighty-one of the 108 samples that were positive in real- time PCR with a Taqman probe produced amplicons in nested PCR of partial regions of the 17kDa, *omp*B and citrate synthase (*gltA*) genes. Sequence analysis of these amplicons revealed the presence of *R. helvetica* in 57 (67%) of the samples. The sequences obtained shared 99–100% similarity with the corresponding gene sequences of *R. helvetica* (GenBank accession numbers AF123725.1 [OmpB], EF392726.1 [17kDa]), and showed significant nucleotide differences from the other rickettsiae in the spotted fever group (Table S1 and S2). Twenty-seven samples reacted positively in real-time PCR, but could not be analysed further because we had no more extracted samples. However, the amplification curves and Ct values for these samples in real-time PCR were similar to samples showing *R. helvetica*, making a presumption of that agent probable. Twenty-four of the samples were found to represent other spotted fever rickettsiae as shown in Table 1 and S2. The partial sequences obtained in 9 of these ticks (7 nymphs and 2 larvae) showed 98–100% similarity with *Rickettsia monacensis* for the *omp*B gene (accession no. EF380356.1) and 98–99% similarity with *R. monacensis* (accession no. 380355.1) or IrTa3 (accession no. AJ427883.1) for the 17kDa gene. In 15 samples, also tested for *omp*B and 17kDa, the partial sequences showed 97–99% similarity with *Rickettsia japonica* (accession no. D16515.1 [17kDa]), *marmioni* (accession no. AY737683.1 [17kDa], *heilongjiangii* (accession no. EU503184.1 [17kDa]) and *heilongjiangensis* (accession no. AY280712.1 [*omp*B]) and for the *omp*A gene, 100% similarity with the *Rickettsia sp.* strain Davousti (accession no. AY280712.1) (Table S2). The interpretations of the individual sequences are shown in Table S2. Some of the 17 kDa sequences were too short to enable species differentiation and for the *R.* sp. strain Davousti, the 17kDa and *omp*B gene sequences are not yet registered in GenBank. When the latter samples, with available extractions, were rerun with primers for the *glt*A and *omp*A genes, the sequence results were in agreement with those found for the o*mp* B gene but with higher similarity for *R*. sp strain Davousti, as shown in Table S2. Samples A41–42, A47–48 and L88–94 came from 3 birds that were infested with more than one infected tick. We have in Table S2 reported sequence variation but the results from samples from the same bird likely represent the same *Rickettsia* sp.

## Discussion

This is the first study to show that migratory passerine birds are an important factor explaining the distribution and expansion of *Rickettsia* spp., including species associated with spotted fever rickettsioses in humans. The birds were equally competent in transmitting rickettsiae to larvae, but they did not seem to serve as reservoir hosts for these agents.

Of the ticks from the single-infested birds, 7.1% were infected with SFR. It is known that ticks can acquire rickettsiae through transovarial passage [1]. The percentage of infected eggs obtained from females of the same tick species varies, and the reason for this is unknown [17], [18]. The presence of rickettsiae in the sub-adult stages could be the result of acquisition through a transovarial route, co-feeding or blood meal acquisition from a rickettsiaemic bird. However, the proportion of birds infested with multiple infected larvae in the present study and the observed counts of infected larvae on individual birds do not exceed the baseline values assumed to represent hypothetical transovarial transmission and do not support the notion that the bird is a source of infection. The higher prevalence among nymphs may result from accumulation of infection in the former stage during consecutive feedings. Ticks are the main vectors and reservoirs of SFG rickettsiae. The role of vertebrate reservoirs in the natural life-cycle of rickettsiae has not been thoroughly examined. Rats, cattle and small mammals have been reported for some SFR species [19]. Many vertebrates are rickettsiaemic only for short periods and in that case ticks are not able to acquire rickettsiae from the bloodstream of their hosts. However, birds are known to be reservoirs for several agents, and the results of the present study do not indicate that this possibility applies to SFR.

In this context, it is interesting to note that later life stages predominated in the *R. monacensis* and *R. heilongjiangensis* samples, whereas approximately half of all *R. helvetica*-positive ticks were *I. ricinus* larvae. An indication of reservoir competence is given in a case with one European Blue Tit (*P. caeruleus*) trapped in autumn that carried seven ticks (six nymphs and a female), of which six were positive for *R. heilongjiangensis*-like *Rickettsia* (Table S2). In this case, it seems likely that the bird was rickettsiaemic and that the ticks had acquired the bacteria from the blood of the bird. This assumption is strengthened by the fact that the bird probably was infested with two *Rickettsia* spp. positive tick species, *I. ricinus* (L88–89) and *I. lividus* (L92–94). Alternatively, bacteria could have been transmitted between co-feeding ticks, given that *I. ricinus* ticks are often aggregated around the beak or the eyes of infested birds. However, there were also several other examples of multiple ticks on a single bird where only one of the ticks was infected with *Rickettsia*, for instance a European Robin that carried 27 ticks, only one of which was positive for *R. helvetica*.

The ground-foraging bird species were 4 times more likely to carry infected ticks than were other species. It is likely that the objective grounds for this difference primarily reflects their feeding habits, as is the case for *Borrelia* sp. and Sindbis virus, resulting in a higher risk of exposure to ticks [15], [20]. There was no difference between the groups when comparing the ratio of birds with infected larvae to the number of larvae-infested birds. In any event, birds that are frequently on the ground contribute effectively to the maintenance and dispersal of the vector ticks and the rickettsial agents.

Most of the ticks were characterized as *I. ricinus*, a tick that has been shown to harbour a few rickettsial species: *R. helvetica*, *R. sibirica* and *R. monacensis* [21], [22]. Fifty-seven (52%) of the infected (108) ticks carried *R. helvetica*, 9 (8.3%) *R. monacensis* and 15 (13.8%) carried a SFR closest to the *R.* sp strain Davousti (Table S2). The ticks were collected from birds on active migration, and knowledge of bird migration ecology could be used to elucidate some aspects of *Rickettsia* biology. For instance, all cases of *R. monacensis*, the etiologic agent of Mediterranean spotted-fever-like illness, were from birds trapped in May, none from the autumn samples. This *Rickettsia* species has previously been described in southern Europe, and among the eight bird species that carried the infected ticks, four were long-distance migrants that winter in sub-Saharan Africa, passing the Mediterranean area and southern Europe during migration, and the remaining four were short-distance migrants where at least part of the population has a winter distribution that partially covers the Western Mediterranean [23].

Fifteen strains of Davousti-like *Rickettsia* were from eight individual birds of five different species, seven from spring and one from autumn. All of these birds were short-distance migrants with wintering ranges mainly in Western Europe. Of particular concern is the finding of six positive ticks from a Eurasian Blue Tit in November, which clearly indicates that this particular Rickettsial species may have an enzootic cycle in the Scandinavian countries.

*R.* strain Davousti has previously been isolated from *Amblyomma tholloni* ticks from elephants, birds and other vertebrates in Africa, and clusters phylogenetically with *R. heilongjiangensis* and *R. japonica* [24]. No *Amblyomma* tick was identified in the present material and *R* sp.strain Davousti has not previously been associated with *Ixodes* spp. ticks. A possible association with *Ixodes* spp. ticks has to be studied further, but it is known that agents may circulate between ticks and hosts in places where different tick species co-exist [25]. *R. heilongjiangensis* has previously been isolated from *Dermacentor silvarum* and *Haemaphysalis consinna* and *R. japonica* from *D. taiwanensis* and *H. flava* [26], [27]. None of these ticks was identified in the present material, and both of these *Rickettsia* spp. have primarily been reported from parts of Asia and Japan. Our findings of these *Rickettsia* spp, in short-distance birds from Sweden expand the knowledge of the known distribution of these agents and motivates further study.

Thus far, *R. helvetica* is the only SFR reported in Sweden, but the influx of new *Rickettsia* spp. into the environment provides opportunities for establishment of new endemic foci with species other than those normally identified. It would therefore seem important to conduct further studies on avian migration patterns to elucidate their influence on disease dissemination as well as on the epidemiology of tick-borne rickettsial agents.

## Materials and Methods

### Bird Capture and Tick Collection

Birds were captured at Ottenby Bird Observatory on the southernmost tip of Öland, a large island in the Baltic Sea (56°12′ N 16°24′ E) south-east of the Swedish mainland. All birds were captured in 2001, throughout the spring (March 17 to May 30) and autumn (July 7 to November 13) migration, in mist nets and Helgoland traps at the observatory as previously described [15], [16]. Every bird was identified by species and examined for ticks daily during these periods, except for 8 days when the excessive number of trapped birds made daily monitoring impossible. Ticks found attached to the bird's head were removed using forceps and placed in individual tubes. The ticks were analysed to identify species and stage and then stored at −70°C until further analysis.

### DNA Extraction, Real-Time PCR

Tick DNA was extracted using Puregene DNA isolation protocol (Gentra Systems, Minneapolis, MN) and stored at −20°C. DNA extracts were assayed for detection of *Rickettsia* spp. using real-time PCR with the probe and primers specific to the *glt*A gene, as previously described [28]. All samples were analysed individually. The PCR was performed in a Lightcycler 1.5 Real-Time PCR System (Roche) using LC Taqman Master kit (Roche). In each reaction, 0.25 µl LC Uracil-DNA glycosylase (UNG) (Roche) was included to minimize the risk of contamination. Sterile water was included as the negative control in each amplification trial. For standards, control DNA was extracted from *R. helvetica* originally isolated from a domestic *I. ricinus* tick [29].

### *Rickettsia* Species Identification and Genotyping

Samples positive in real-time PCR were further analysed using two nested PCR assays that amplify partial sequences of the 17-kDa and *omp*B genes, as previously described [30], [31]. For some of the samples, two additional PCR assays representing the *glt*A gene and *omp*A genes were used as well (Table 2) [32], [33]. Amplification was carried out in a DNA thermal cycler (MJ Mini Gradient Thermal Cycler, Bio-Rad Lab, California) and expected fragment sizes were confirmed using gel electrophoresis (2% agarose). Confirmation of fragment size was based on a standard DNA molecular weight marker (Invitrogen). Negative and positive controls were included in each PCR run. Purified DNA of *R. helvetica* originally isolated from a domestic *I. ricinus* tick was used as the positive control [29]. Direct cycle sequencing analysis of both strands of nested PCR products was performed at the Center for Genomics and Bioinformatics, KI, Stockholm (KI Gene), and pairwise similarities to and differences from other rickettsiae in the spotted fever group were examined using Blast analysis. Sequence alignments were performed using BioEdit version 7.0.9.

**Table 2:** Details of selected inner primers and probe used to amplify rickettsial *omp*B, *omp*A, 17kDa, and *glt*A genes.

Primer	Gene	Nucleotide sequences (5′ to 3′)	Product size (bp)
OMP B–IF	*omp*B	CCA-ATG-GCA-GGA-CTT-AGC-TAC-T	267
OMP B-IR		AGG-CTG-GCT-GAT-ACA-CGG-AGT-AA	
Rr190.70p	*omp*A	ATG-GCG-AAT-ATT-TCT-CCA-AAA	590
190.701		GTT-CCG-TTA-ATG-GCA-GCA-TCT	
RH 17-IFRH	17 kDa	GCA-TTA-CTT-GGT-TCT-CAA-TTG-G	214
17-IR		AAC-CGT-AAT-TGC-CGT-TAT-CCG-G	
Rp877-F	*glt*A	GGG-GGA-CCT-GCTCAC-GGC-GG	381
Rp1258-R		ATT-GCA-AAA-AGT-ACA-GTG-AAC-A	
CS535-F	*glt*A	GCA-ATG-TCT-TAT-AAA-TAT-TC	355
CS890-R		GCT-TTA-GCT-ACA-TAT-TTA-GG	
SFG-CS-F	*glt*A	TGC-CAA-ATG-TTC-ACG-GTA-CTT-T	74
SFG-CS-R		CAC-AAT-GGA-AAG-AAA-TGC-ACG-A	
SFG-CS-P	*glt*A	TGC-AAT-AGC-AAG-AAC-CGT-AGG-CTG-GAT-G	probe

### Statistical Analyses

For continuous variables, we used standard parametric statistics giving a mean±95% confidence interval (CI). For proportions we used Fisher's exact test, Chi square goodness of fit, and odds ratio (OR) procedures. Statistical analyses were conducted using Predictive Analytics Software (PASW®) version 17.0.2.

### Ethics Statement

Trapping of birds was approved by the National Ringing Centre at the Swedish Museum of Natural History, Stockholm, and by the board of Ottenby Bird Observatory. Ethical approval for sampling ticks from birds was obtained from the Malmö/Lund animal research ethics board.

## Supporting Information

Table S1 Summary of bird species infested by *Ixodes ricinus* ticks, number of tick-stages and rickettsia DNA positive tick.
(0.03 MB XLS)

Table S2 Results of PCR and alignment of amplified partial sequences for 17 kDa, ompB and ompA genes for 81 rickettsial DNA tick samples. The score for similar identities or other less similar alternatives are shown in footnotes for most of the sequences.
(0.03 MB XLS)
